# Strengthening through adversity: The hormesis model in developmental psychopathology

**DOI:** 10.1017/S0954579424000427

**Published:** 2024-03-27

**Authors:** Assaf Oshri, Cullin J. Howard, Linhao Zhang, Ava Reck, Zehua Cui, Sihong Liu, Erinn Duprey, Avary I. Evans, Rabeeh Azarmehr, Charles F. Geier

**Affiliations:** 1 Department of Human Development and Family Science, University of Georgia, Athens, GA, USA; 2 Department of Psychology, University of Maryland, College Park, MD, USA; 3 Graduate School of Education, Stanford University, Palo Alto, CA, USA; 4 Department of Psychology, University of Rochester, Rochester, NY, USA

**Keywords:** adversity, brain-by-developmental context, default mode network (dmn), equifinality, hormesis, multifinality, neuroplasticity resting-state functional connectivity, steeling, resilience

## Abstract

**Background:**

Employing a developmental psychopathology framework, we tested the utility of the hormesis model in examining the strengthening of children and youth through limited levels of adversity in relation to internalizing and externalizing outcomes within a brain-by-development context.

**Methods:**

Analyzing data from the Adolescent Brain and Cognitive Development study (*N* = 11,878), we formed latent factors of threat, deprivation, and unpredictability. We examined linear and nonlinear associations between adversity dimensions and youth psychopathology symptoms and how change of resting-state functional connectivity (rsFC) in the default mode network (DMN) from Time 1 to Time 5 moderates these associations.

**Results:**

A cubic association was found between threat and youth internalizing problems; low-to-moderate family conflict levels reduced these problems. Deprivation also displayed a cubic relation with youth externalizing problems, with moderate deprivation levels associated with fewer problems. Unpredictability linearly increased both problem types. Change in DMN rsFC significantly moderated the cubic link between threat levels and internalizing problems, with declining DMN rsFC levels from Time 1 to Time 5 facilitating hormesis. Hormetic effects peaked earlier, emphasizing the importance of sensitive periods and developmental timing of outcomes related to earlier experiences.

**Conclusions:**

Strengthening through limited environmental adversity is crucial for developing human resilience. Understanding this process requires considering both linear and nonlinear adversity-psychopathology associations. Testing individual differences by brain and developmental context will inform preventive intervention programming.

## Prologue

Developmental psychopathology (DP) is a system perspective on human development, focusing on the origins and course of individual patterns of behavioral adaptation and maladaptation. DP considers these patterns within the context of normal versus abnormal developmental processes while considering typical and atypical developmental processes and outcomes (Cicchetti & Toth, 2009). Dr Dante Cicchetti, a pioneer in the field, has made numerous substantial theoretical and empirical contributions to the field of developmental psychopathology. Notably, Dr Cicchetti’s research and theory have addressed heterogeneity in developmental outcomes and advocated multilevel research approaches (Rutter, 2006, 2012). In the words of Sir Rutter (2008), “Cicchetti (1990) similarly emphasized the need to focus on causal processes but went further in insisting that the scientific enterprise had to incorporate genetics and neuroscience as well as psychological studies. One particular contribution of his was the emphasis on the need to recognize the diverse pathways that could lead to the same endpoint….” The present paper will utilize these advances in developmental psychopathology to delve into heterogeneity in human resilience. We adopt a multilevel approach to study the nonlinear brain and behavioral mechanisms of human resilience to environmental adversity.

In the past 30 years, developmental scientists have reached a consensus that resilience is a dynamic process in which, despite significant adversity, children and youth benefit from protective factors (e.g., assets and resources) over time and follow positive trajectories across multiple functioning domains (Curtis & Cicchetti, 2003; Luthar & Zelazo, 2003; Masten, 2001). The study of resilience is an integral part of the DP paradigm (Cicchetti, 2010; Masten et al., 1990; Rutter, 2012). Indeed, DP research has been transformative in advancing resilience science by focusing on continuities and discontinuities in human development over time (Cicchetti, 2010; Masten et al., 1990; Rutter, 2012). Increasingly, scholars interested in resilience research have shifted their investigations to a multilevel and multimethod approach, modeling risk and protective factors mediating and moderating resilience processes. Developmental psychopathology tenets, including equifinality and multifinality, have influenced these multimethod and multilevel approaches. The idea that organisms, including humans, who were exposed to similar adverse environments may follow diverse developmental trajectories and outcomes has been referred to as the concept of multifinality (Beauchaine et al., 2009; Cicchetti & Rogosch, 1996; Von Bertalanffy, 1972). Conversely, the idea of equifinality suggests that individuals exposed to different environmental conditions might, over time, exhibit similar outcomes (Cicchetti & Rogosch, 1996). Recent advances in methodological and statistical tools have enabled researchers to conduct rigorous research informed by these concepts of equifinality and multifinality. For example, in longitudinal systems models, risk and protective factors transact to produce homogeneous and heterogeneous outcomes in development after exposure to environmental adversity (Cicchetti & Toth, 2009; Masten & Cicchetti, 2016).

## Prevailing limitations in the study of human resilience

Despite scientific progress in studying human resilience using a developmental lens, some significant methodological issues remain to be addressed (Curtis & Cicchetti, 2003; Oshri et al., 2018). These issues hamper progress in answering questions about the nature of resilience in human development. A salient and prevailing question is why psychosocial adversity affects the development of some and not others. In other words, what explains the heterogeneity of developmental outcomes in response to psychosocial adversity? First, stressors or adverse experiences encompass complex environmental conditions that are often difficult to operationalize in the context of a research study (Cicchetti & Toth, 2016; Nikolaidis et al., 2022). Human development researchers focusing on risk and resilience processes have recently proposed a dimensional approach to typify adversity (Berman et al., 2022; McLaughlin & Sheridan, 2016). This growing body of research has shown that ignoring the dimensionality of environmental adversity impedes knowledge of the developmental etiology of human risk and resilience (LoPilato et al., 2020; McLaughlin et al., 2014; Nikolaidis et al., 2022).

Second, exposure to adversity may promote adaptation to the environment and even resilience to adverse contexts (Ellis et al., 2009; Fenneman & Frankenhuis, 2020). However, not every adverse environment type, severity, or developmental timing may trigger strengthening effects. We suggest that modeling linear models on the impact of adverse life environments on human development outcomes may miss nonlinear salubrious and protective effects (Davies et al., 2022; Oshri et al., 2022; Oshri, 2023). For example, over time the process of resilience may emerge *in response* and not despite exposure to specific types or certain levels of adversity (i.e., strengthening effects). These strengthening effects are understudied and must be further explored, addressed, and better understood via nonlinear modeling.

Lastly, although strengthening effects are evident in response to some types and severities of adversities, there are substantial interindividual differences in when and who benefits from these promotive effects. Modeling human resilience development necessitates investigating the variability-inducing contexts that promote strengthening effects (Riley & Masten, 2005; Wyman, 2003). Though not exhaustive, these issues and limitations highlight significant challenges in resilience science that persist despite using current longitudinal multilevel multimethod and system approaches. The present study aims to address these limitations and contribute to a new generation of research on the developmental mechanisms of human resilience in the field of developmental psychopathology.

Adopting a DP perspective, the present study aims to examine the dual role of adversity in the development of psychopathology, highlighting both risk and resilience effects. This approach seeks to enrich and guide future resilience research anchored in developmental psychopathology principles. To tackle the complexity of measuring adversity, we will employ a dimensional approach to assess early adversity (Ellis et al., 2009; McLaughlin & Sheridan, 2016; McLaughlin et al., 2014). This method involves categorizing adversity into a taxonomy that distinguishes environments based on experiences of deprivation, threat, and unpredictability (Ellis et al., 2009; McLaughlin & Sheridan, 2016; McLaughlin et al., 2014).

## The hormesis model and developmental psychopathology

**Figure 1.**
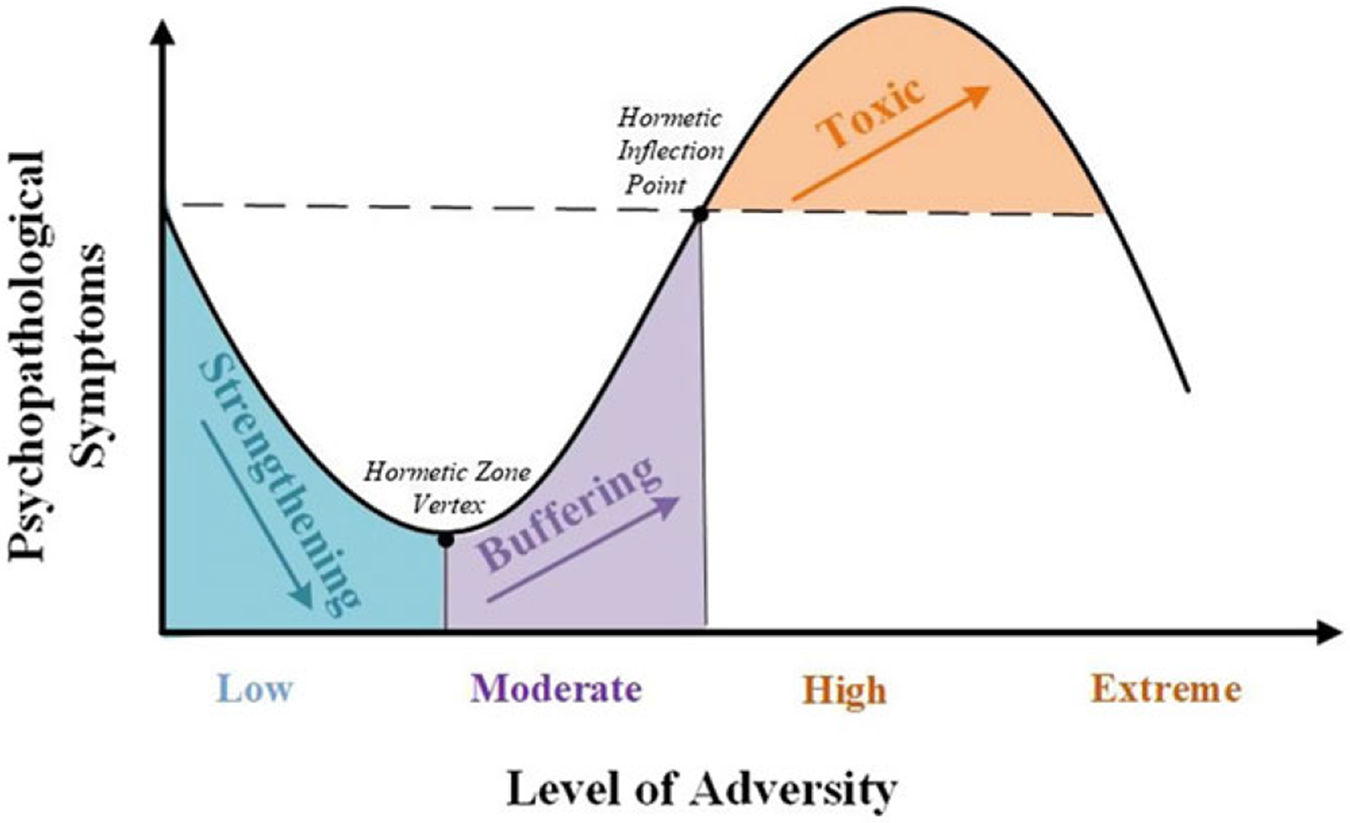
The psychosocial hormesis model.

The theoretical model we will use to examine the nonlinear and dynamic response to adversity elucidates the phases of strengthening effects from circumscribed psychosocial hardship (Fig. 1). The hormesis model has been used in other disciplines for over a century (Calabrese & Baldwin, 2000). We will ground our investigation on the *psychosocial stress model of hormesis,* a refined and adapted theoretical version of hormesis (Oshri et al., 2022), to study the emergence of the resilience process under varying levels of environmental stressors and developmental contexts (Oshri et al., 2022; Oshri, 2023). According to this model, low-to-moderate levels of adversity may lead to reduced risk up to an inflection point, after which higher levels of adversity lead to risk for psychopathology (Oshri et al., 2022; Oshri, 2023). Moreover, these nonlinear effects cover different aspects of behavioral outcomes to varying levels or severity of psychosocial adversity. Lastly, as neuroplasticity declines with age, we expect that hormetic effects on risk for psychopathology will be more salient in younger youth. Using linear, quadratic and cubic functions, we will showcase how this can be identified and probed in longitudinal modeling.

Figure 1 provides a diagrammatic presentation of the theoretical mechanisms proposed in Oshri et al. (2022). The overall curve can be classified into two zones (hormetic and toxic) that are separated by the hormetic inflection point at which adversity no longer confers a degree of benefit but is deleterious. The hormetic zone is further divided into two regions (strengthening and buffering) by the hormetic zone threshold, or vertex (i.e., the highest or lowest point of a quadratic function). The “strengthening effect” (the area on the left of the hormetic zone vertex in Fig. 1) is characterized by an adaptive response system underlain by a neurobiological mechanism (referred to here as *hormetic neuroplasticity*) that prepares or conditions the individual for future environmental adversity. The “buffering effect” (on the right side of the hormetic zone vertex) starts when the adversity level reaches a juncture (stress threshold) and changes direction in relation to psychopathological outcomes. The hormetic zone vertex is defined by the maximum level of adversity in which strengthening occurs (as measured by reduced risk for psychopathology) and the beginning of decreased adjustment benefits following exposure to higher levels of adversity. The latter region is referred to as buffering (and belonging to the hormetic zone) because it includes individuals who display a lower risk for psychopathology than those who have none to minimal levels of adversity. Beyond the hormetic inflection point is the toxic stress zone, and is distinguished from the hormetic zone. This area of extreme stress has been discussed in various models of stress on development (Shonkoff et al., 2012). Adversity levels in the toxic zone are thought to have an overwhelmingly negative impact on the individual and induce risk for the development of psychopathological outcomes.

This study will investigate individual differences in hormetic responses by analyzing (1) the nonlinear associations between dimensions of adversity and symptoms of psychopathology and (2) considering interindividual differences in hormetic effects across changes within a brain network connectivity context that is dynamic and reflects neuroplasticity potential over time. This approach recognizes that environmental and developmental context matters, and not every individual benefits equally at any given time from any given type of limited exposure to adversity (Oshri et al., 2022). Therefore, we aim to delineate the brain processes contributing to this heterogeneity in hormetic responses. In particular, this examination will highlight how individual variability in responding to adversity can be partially attributed to the evolving neuroplastic processes in the brain, especially those related to self-regulation, future orientation, and learning.

Data in the present study are drawn from the Adolescent Brain and Cognitive Development (ABCD) project, an existing dataset considered the largest longitudinal neurobiological study of youth development in history. Given the ABCD’s assessment limitation of environmental context, we will leverage structural equation modeling (SEM) to latently measure environmental adversity dimensions optimally. Without experimental data, attention to measurement timing (across assessment time points) enables us to keep fidelity to methods that ensure proximity to causal inference between independent, dependent, and moderating variables. Therefore, we will prioritize the temporal order of causation to examine causal effects in these longitudinal, multilevel, multimethod models.

## Dimensional adversity and risk for youth psychopathology

### Threat

Threat is defined as experiences involving or risking harm (McLaughlin et al., 2014). This study will focus on threatful environments involving conflict in the family context. Children’s exposure to family conflict has been conceptualized as an early form of a threatening rearing environment (Davies et al., 2021; Fosco & Feinberg, 2015). Extant research shows that children raised in families laden with family conflict, such as parent-child, interparental, and sibling conflict, are at risk for a range of problem behaviors (Grych et al., 2000). This has led developmental scientists to formulate the emotional security theory (Cummings & Davies, 2010; Davies & Cummings, 1994), whereby children experience unsafe situations due to family conflict. This theory suggests family conflict leads to vulnerability for later psychopathology due to the development of dysregulated response patterns to threatening environments (Cummings & Davies, 2010; McLaughlin et al., 2014). Data on the strengthening effect of adversity in response to threats such as family conflict exist. In a prospective study, Davies et al. (2022) showed a nonlinear (i.e., quadratic) association between children’s emotional reactivity and behavioral dysregulation. Specifically, the study showed that minimal levels of family conflict were linked to less emotional risk, whereas high levels of family conflict were related to children’s increased risk for psychopathology. Based on this previous research, we expect a hormetic effect in response to low-to-moderate threat levels.

### Deprivation

Deprivation experiences include an absence of expected inputs from the environment (McLaughlin & Sheridan, 2016). Children reared in families who endure material hardship are at risk of experiencing multiple sources of deprivation, scarce financial support, limited access to neighborhood and community resources, and reduced parental supervision (Heflin et al., 2009). Socioeconomic (SES) hardship is a robust predictor of adolescent risk behaviors and a range of adjustment problems throughout adolescence and early adulthood, including psychopathology and addiction (Bolland et al., 2016; Lopez-Vergara et al., 2016). Similarly, when it comes to parenting, there is robust evidence that depriving attention and awareness of the child’s whereabouts is linked to problem behaviors, particularly delinquency (Corlis & Damashek, 2021).

Despite robust evidence linking deprivation to the risk of psychopathological outcomes, this association may exhibit a nonlinear pattern indicative of hormesis. For instance, during the transition to adolescence, parental monitoring and control levels diminish as children seek individuation and increasingly spend time with peers. Furthermore, during this developmental transition, neglectful parenting practices evolve from a lack of parental supervision to a lack of parental monitoring (Burke et al., 2008; Ryan et al., 2013). At this stage, some reduction in parental monitoring could positively influence the development of autonomy in youth. Because a critical developmental task in adolescence is to develop autonomy, it is possible that in a normative sample, some degree of lack of parental monitoring coupled with financial distress can lead to the development of valuable life skills, such as self-reliance, problem-solving abilities, and coping strategies. These life skills are essential for resilient adolescents (Zimmer-Gembeck & Skinner, 2016) and may result in the development of strengthening and coping skills. Further, a nonlinear effect is also expected in response to SES hardship, challenging the idea that higher SES necessarily leads to favorable developmental outcomes. In fact, growing research shows that privileged children reared in affluent and resourceful environments are also at risk for psychopathology (Luthar & Latendresse, 2005). It is plausible, therefore, that exposure to some level of economic hardship will trigger youth to develop essential life skills that will help them negotiate stressors in life. Consequently, we hypothesize a hormetic effect in the link between deprivation and risk for the development of psychopathology.

### Unpredictability

Unpredictability is a less-studied form of environmental adversity in relation to the development of psychopathology. Defined as conditions at which environmental harshness varies over time and space, developmental science research often refers to inconsistent rates and unreliable rearing environments (Ellis et al., 2009). A core feature of child development is children’s ability to learn and be shaped by the environment (Ferguson et al., 2013). This is evident in social learning and the underlying neural mechanisms linked to neuroplasticity (Davidson & McEwen, 2012). Neural organization and proliferation in response to environmental input are often mediated via two types of organism-by-environment interactions: experience-*expectant* and experience-*dependent* (Schore, 1996). Learning via experience-expectant input involves neural responses after exposure to specific expected input during sensitive and critical periods. For example, there is a window of language acquisition among infants and young children largely due to early-life neuroplasticity and the unique ability of infants and young children to distinguish between all phonemes (distinct units of sound).

In contrast, experience-dependent is more pertinent to hormetic effects as it refers to any neuroplastic learning experience spanning the lifespan, such as learning how to drive a car, or even coping strategies such as reappraisal to promote emotion regulation (Gabard-Durnam & McLaughlin, 2019; Greenough et al., 1987). Unpredictability in the environment constitutes a salient stressor for the child because environmental input is stochastic, which hinders the learning processes necessary for hormesis. The life history perspective suggests that stress linked to unpredictability triggers survival strategies that promote earlier reproductive behaviors and the propagation of genes. Indeed, unpredictability-related stress has negatively influenced development and motivation in animal and human research (Chen & Baram, 2016; Xu et al., 2023). Growing developmental psychopathology research shows that unreliability is a robust predictor of children and youth externalizing and internalizing psychopathology (Glynn & Baram, 2019; Hunt et al., 2023). Because an unpredictable environment offers minimal opportunity for learning and is associated with mental health risks, we didn’t hypothesize a strengthening effect from experiences of unpredictability among youth (Glynn & Baram, 2019; Hunt et al., 2023).

## Development in brain context: the case of resting-state functional connectivity of the DMN

Ecological perspectives DP (Cicchetti, 2008; Hyde et al., 2020) suggest that the degree to which environmental adversity affects psychopathology may vary by neurobiological contexts (Boyce, 2016). The present study focuses on the neural context of functional connectivity (i.e., between-region correlated brain activity) within the default mode network (DMN) derived from resting-state fMRI (rs-fMRI) data acquired as part of the ABCD Study (Casey et al., 2018). We deliberately focused on brain networks instead of specific regions of interest (Bassett & Sporns, 2017; Pessoa, 2014). A network approach to understanding brain function reflects the interconnected and dynamic nature of the brain, offering a more accurate and comprehensive view than examining isolated regions alone (Bassett & Sporns, 2017; Pessoa, 2014). This perspective is crucial for advancing the discovery and understanding of the neural substrates underlying psychiatric disorders as well as complex (Bassett et al., 2018), emergent brain functions more generally. Growing empirical evidence suggests that neuroregulatory capacities are often linked to interconnectivity and communication between multiple regions of interest forming networks. These neural networks better predict specific reactions above and beyond the effects of any singular neural region (Menon, 2011; Pessoa, 2014).

We selected to examine the DMN because of its functions related to resilience and psychopathology. The DMN is one of several large-scale brain networks identified during “rest” – when the brain is awake but not otherwise engaged in task-related activity (Broyd et al., 2009; Tashjian et al., 2017). Reported functions supported by DMN include self-referential thought and recalling personal memories, daydreaming, social cognition, and introspection (Andrews-Hanna et al., 2014; Wade et al., 2018). The DMN also regulates attention and switches between internal and external modes of attention (Whitfield-Gabrieli & Ford, 2012). Resting-state networks such as the DMN offer a window into the brain’s intrinsic functional organization and architecture, as these networks are constructed from a history of coactivation between brain regions (Greicius et al., 2009). Research shows that transactions between a person’s unique experiences and context and their maturing neural substrate shape these networks across time (Sripada et al., 2014; Wang et al., 2019). These changing and multilevel interactions (brain-by-development) explain the emergent properties of brain network development, which contextualize human resilience as a dynamic process that emerges from the interaction between the environment and individual neurobiology. From this perspective, changes in resting-state networks are critical context for the neuroplasticity processes expected in adolescence. The network level resting-state functional connectivity (rsFC) is a malleable asset the child has developed and continues to develop through their experiences (DeCross et al., 2022; Whittle et al., 2017).

Neuroimaging studies, primarily those using rs-fMRI, have identified that the DMN consists of a distributed set of brain regions, including the medial prefrontal cortex, posterior cingulate cortex, precuneus, and lateral parietal cortex, among others (Broyd et al., 2009). The level of connectivity between all brain regions comprising the DMN is called intra-network connectivity (hereafter called DMN rsFC), operationalized here as the mean correlation value across all connections between DMN regions defined in the Gordon et al. (2016) i.e., DMN-to-DMN connections. Notably, within-DMN activation changes during adolescence due to normative maturational processes (Horowitz-Kraus et al., 2017). In line with a developmental psychopathology systems framework (Cicchetti & Curtis, 2015), large-scale brain networks like the DMN can and should be evaluated across multiple levels of analysis to better understand how different brain regions interact and communicate.

The dynamic interaction between the environment, DMN rsFC, and risk for psychopathology versus resilience is only beginning to be understood (Yaesoubi et al., 2015). The DMN has been linked to behavioral outcomes involved in resilience among youth, such as future orientation (Fuentes-Claramonte et al., 2019), cognitive inhibition (Fulong et al., 2020), and coping with psychosocial stress and adversity (Rebello et al., 2018). Some research suggests that DMN rsFC patterns are associated with better emotion regulation and resilience (Miyagi et al., 2020). However, other results indicate relative hyperconnectivity may also be linked to rumination and risk for psychopathology (Whitfield-Gabrieli & Ford, 2012). Further, studies examining the association between DMN connectivity patterns and impulsivity have yielded competing evidence. For instance, Inuggi et al. (2014) showed that higher trait-impulsivity is related to lower DMN rsFC among 8 to 12–year–olds. Further, risk-taking behaviors in adolescents have been associated with DMN hyperconnectivity during resting state (DeWitt et al., 2014), suggesting that stronger DMN rsFC could contribute to youths’ impulsivity. Lastly, DMN rsFC has been associated with adaptive socioemotional functioning in adolescence, including sleep health (Tashjian et al., 2018; Zhang et al., 2023), emotional regulation(Afzali et al., 2022) and reduced problem behaviors (Fox et al., 2017). Given these functional implications, we examined the DMN rsFC of this network as a neural context where different dimensions of adversity are differentially hormetic and consequently linked to variability of risk for the development of psychopathology.

## Developmental timing of hormetic plasticity

Although a large body of research shows that there are sensitive developmental periods to the negative impact of adversity (Knudsen, 2004), less is known about sensitive periods for strengthening through adversity effects. Similarly, existing literature indicates that neuroplasticity diminishes with age (Gabard-Durnam & McLaughlin, 2019). Consequently, it is reasonable to hypothesize that stress-induced neuroplasticity will be differentially expressed in relation to different timings of developmental outcomes (Knudsen, 2004). This concept suggests that the neurobiological processes underlying hormetic effects are more pronounced during sensitive periods. Exposure to low-to-moderate levels of adversity may facilitate experience-expectant and dependent learning, potentially serving as an asset to mitigate the impact of future adversity later in development. This phenomenon of hormetic neuroplasticity timing, or the brain’s adaptive response to stressors, may provide a foundational mechanism for resilience in adverse environments. Therefore, longitudinal studies investigating hormetic effects at varying developmental stages and ages are crucial to elucidate the brain mechanisms driving resilience. We posit that part of individual differences in hormetic effects is correlated with time-varying experience-dependent neuroplasticity.

## The present study

The main aim of the present study is to examine to what extent and under what adverse conditions children may show linear risk for psychopathology and strengthening effects in response to adversity using the psychosocial hormesis theoretical model (Oshri, 2023). Environmental adversity will be tested across three dimensions: threat, deprivation, and unpredictability. We first hypothesized that the three dimensions of psychosocial adversity will be linearly associated with increased internalizing and externalizing symptoms. We did not have a preconceived hypothesis on expected hormetic effects in response to unpredictability. Given previous literature, however, we did expect a nonlinear effect–that low-to-moderate levels of deprivation and family threat would be consistent with hormesis. In line with the psychosocial hormesis model, we tested the hypothesis that DMN rsFC would serve as a developmental context contributing to variability in hormetic response. Specifically, we examined whether individual differences in hormetic effects could be explained by varying levels of change in DMN rsFC and the age in which the hormetic outcomes are modeled. We hypothesized that age of change in psychopathological risk symptoms (developmental timing) and alterations in DMN rsFC patterns would differentiate youth who benefit from low-to-moderate adversity levels. The aim was to test the hypothesis that changes in brain networks, such as changes in within-DMN rsFC, can lead to differences in response to low-to-moderate stress exposure and inform prevention intervention programs.

## Methods

### Sample

All hypotheses were tested using data from the ongoing ABCD study, a longitudinal and multisite study of adolescent brain development and mental health. The study included a total of 11,878 children from 21 sites across the United States, representing diverse socioeconomic, ethnic, and biobehavioral health backgrounds. The study procedures received approval from the human research protection programs and institutional review boards at all universities participating in the ABCD project. Full details on study aims, design, recruitment, and procedures may be found in Casey et al. (2018) and Garavan et al. (2018). Data accessed from the NIMH data included baseline (*N* = 11,878; 47.8% female; *M*_age_ = 10.1), T2 (6 months from baseline), T5 (24 months from baseline), and T7 (36 months from baseline, *M*_age_ = 12.9). The racial-ethnic composition of the sample was 52.0% European American, 15.0% African American, 20.3% Latino(a), 2.1% Asian/Pacific Islander, and 10.5% Other.

### Resting-state functional magnetic resonance imaging data

Neuroimaging data were obtained using Siemens, General Electric, or Philips 3T scanners equipped with a 32-channel head coil. During the collection of rs fMRI data, participants were instructed to focus on a crosshair for 20 minutes. The preprocessing of fMRI data utilized FreeSurfer version 5.3.0 before data extraction (Casey et al., 2018) and by the ABCD Data Analysis and Informatics Core (Hagler et al., 2019). We then followed the recommended quality control protocol (Hagler et al., 2019) and excluded participants exhibiting excessive head motion. Complete imaging data remained for 9,130 participants at T1 and 7,194 at T5.

### Measures

#### Predictors

##### Family threat.

A latent factor comprising seven items from the Family Environment Scale family conflict subscale was used to assess family threat (Hoffman et al., 2019; Zucker et al., 2018). All items were coded in the same direction, and the factor structure was optimized via confirmatory factor analysis (CFA; see Table 1 for items and factor loadings, *χ*^2^(12) = 444.27, RMSEA = .06, CFI/TLI = .96/.93, SRMR = .06.)

##### Deprivation.

A latent factor of SES-related deprivation was created using a CFA at T1, including caregivers’ marital status (1 = no, 0 = yes), family material deprivation, parental education (1 = < HS Diploma….5 = Post Graduate Degree), family income-to-poverty ratio (dividing the family’s reported annual income by the federal poverty threshold for the year, accounting for household size), neighborhood area deprivation index, and parental monitoring. Family material deprivation was measured via a total of seven items from the Parent Demographics Survey that assessed whether (1 = yes, 0 = no) a family experienced any financial-related hardships (e.g., could not afford food) during the past 12 months. The area deprivation index is a composite score derived from the American Community Survey, incorporating data on 17 neighborhood factors (e.g., income, education, employment, and housing quality). Parental monitoring is measured by one question regarding the frequency of shared dinners between parents and the participant in an average week (Table 1). The model fit for the factor structure was good, *χ*^2^(9) = 54.60, RMSEA = .02, CFI/TLI = 1.00/.99, SRMR = .01.

##### Unpredictability.

The degree of unpredictability within the family was conceptualized as the combination of unpredictable or chaotic behaviors manifested from primary/secondary caregivers, and the amount of disorder characterizing the family environment. This was operationalized through a higher-order CFA composed of primary caregiver unpredictability, secondary caregiver unpredictability, and family disorder as first-order factors. Items for each of the first-order factors were taken from the Adult Behavior Checklist (Achenbach et al., 2003) and the Family Environment Scale (Moos & Moos, 1994), respectively (See Table 1 for items and factor loadings). The model fit for the higher-order factor structure was good, *χ*^2^(31) = 355.73, RMSEA = .03, CFI/TLI = .967/.952, SRMR = .03.

##### Within-DMN rsFC change.

Network connectivity within-DMN (Pearson correlation) was calculated based on the Gordon parcellation scheme (Gordon et al., 2016) for 12 predefined resting-state networks, including the DMN. Imaging was completed at baseline (T1) and the two-year assessment (T5). A single-indicator latent change score was computed and saved to represent the degree of change in within-DMN rsFC from T1 to T5 (ΔDMN rsFC). In neurocognitive developmental studies, latent change scores are recommended for characterizing neural change over time (Kievit et al., 2018). Positive ΔDMN rsFC scores indicate the average correlation across all regions within the DMN network increasing from T1 to T5 (i.e., greater DMN rsFC over time), while negative ΔDMN rsFC scores indicate decreases in DMN rsFC over time. On average, the overall sample was characterized by increases in DMN rsFC from T1 to T5, with significant variability (μΔ_DMN-rsFC_ = .25, *σ*^2^_DMN-rsFC_ = .003). The latent change model for ΔDMN rsFC was just identified.

### Outcome measures

#### Internalizing and externalizing problems

Adolescents self-reported their internalizing and externalizing symptoms using 19 items from the Brief Problem Monitor (BPM) (Achenbach et al., 2011). BPM was administered every 6 months from T2 to T7. Items were assessed on a Likert scale ranging from “0” (*not true*) to “2” (*very true*). The internalizing problems subscale (α_T2_ = .71; α_T5_ = .78; α_T7_ = .80) reflects a child’s emotional and internal psychological state and the externalizing problems (α_T2_ = α_T1_ = .67; α_T5_ = .67; α_T7_ = .67) capture a child’s problem behavior that is directed toward the external environment. Single-item latent change scores were computed using the summary score data from T2 - T7 (Geiser, 2020). Model fit indices were good for both the internalizing, *χ*^2^(9) = 56, RMSEA = .02, CFI/TLI = 1.00/.99, SRMR = .02, and externalizing, *χ*^2^(9) = 36.74, RMSEA = .02, CFI/TLI = 1.00/.99, SRMR = .02 models.

#### Covariates

Youth sex (1 = female, 2 = male), income (<5K = 10, …,> = 200K = 1), and parental education (1 = < HS Diploma….5 = Post Graduate Degree) were included as covariates.

### Analytic plan

Hypotheses were tested using SEM in Mplus version 8.1 (Muthén & Muthén, 2009), and figures were generated using the Rstudio version 4.2.0 (R Core Team, 2022). The factor structure of the adversity latent variables (family threat, deprivation, and unpredictability) was assessed through CFA, factor scores saved and mean-centered at zero. Second, linear and nonlinear longitudinal associations between each dimension of adversity and change in later youth behavioral problems were examined using path analysis. Significant nonlinear effects were probed for hormetic patterns by plotting the slope, calculating inflection points/vertices (i.e., values partitioning the cubic curve in conceptually meaningful sections), and estimating the number of participants characterized by each region. Hormetic and toxic zones were distinguished by the *conceptual* hormetic inflection (where predicted buffering region values surpassed the predicted strengthening region values), rather than the *statistical* hormetic inflection (pivot point in curve concavity). Both points have been identified on cubic plots for interested readers. Last, latent change in DMN rsFC (from T1 to T5) was tested as a moderator of cubic hormetic effects. Simple slopes of significant cubic moderations were plotted and further probed using multi-group linear/quadratic interaction analysis parceled by region/zone. Covariates were included in all analyses. Multilevel modeling was used to account for the clustering effects of participants within families and scanner type.

A full-information maximum likelihood algorithm was used to estimate the missing data. The weighted least squares with mean and variance adjusted estimator was used for estimating adversity latent factors because of the presence of nominal indicators within the measurement model. Participant factor scores derived from CFAs (including latent change scores) were saved to facilitate their usage in subsequent analyses and transformation to nonlinear regression terms. All other analyses used maximum likelihood estimation with robust standard errors as it produces unbiased parameter estimates (Enders & Bandalos, 2001). Criteria for evaluating model fit were as follows: a maximum value of .06 for the root mean square error of approximation (RMSEA) and .08 for the standardized root mean squared (SRMR), and a minimum value of .90 for the comparative fit index (CFI) (Hu & Bentler, 1999).

## Results

### Linear and nonlinear effects of adversity on youth outcomes

Youth age, sex, and family income significantly predicted change in youth internalizing and externalizing problems (Table 2). Nonsignificant covariates (e.g., primary, and secondary parental education) were excluded from further models.

### Family threat

**Figure 2.**
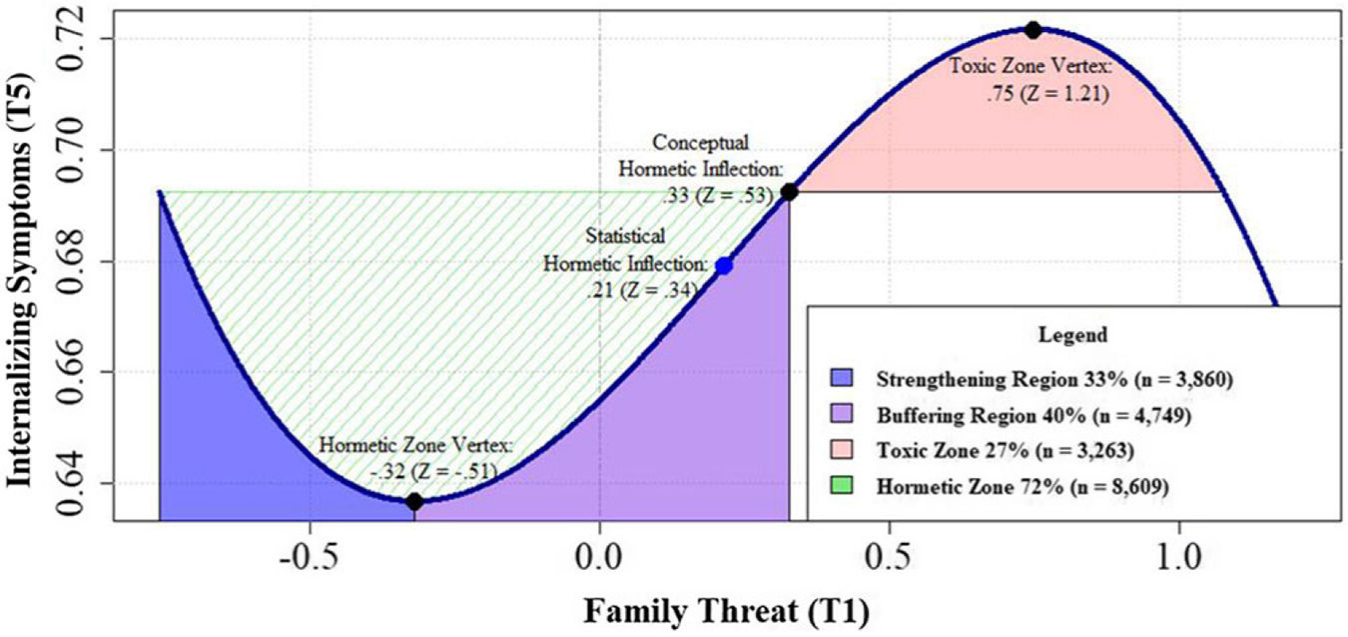
Probing hormesis of family threat effect on internalizing symptoms.

We first tested a linear, quadratic, and cubic SEM model on the predictive associations between the level of family threat and internalizing and externalizing symptoms. The linear results showed that family threat predicts significant increases in externalizing problems at both T5, *β*_threat_ = .08, *CI*[.06, .11], *p* < .001 and T7, β_threat_ = .03, *CI*[.01, .05], *p* < .05, but not in internalizing problems at either time point. While the quadratic model was largely nonsignificant, adding the cubic family threat path significantly improved model fit, LRT: Δ*χ*^2^(4): 9.68, *p* < .05, and this model predicted changes in internalizing *and* externalizing problems. In particular, the linear family threat path persisted in predicting youth externalizing problems at T5 and T7 and the cubic family threat term predicted internalizing problems at T5, *β*_threat_^3^ = −.07, *CI*[−.13, −.01], *p* < .05. Follow-up analyses indicated that the conceptual hormetic inflection point (i.e., the transition from hormetic to toxic adversity zones) of the cubic curve occurred at .33 units above the mean (*Z* = .53, see Fig. 2). Within the hormetic zone, the hormetic zone vertex (i.e., the transition from strengthening to buffering adversity regions) for family threat is at −.32 units below the mean (*Z* = −.52). Using these thresholds, the majority of the youth in the total sample are characterized by the hormetic zone, either in the strengthening (33%, *n* = 3,860) or buffering (40%, *n* = 4,749) regions. In contrast, 27% of the sample (*n* = 3,263) experienced family threat characteristic of the toxic zone. Overall, this model explained 2% of the change in externalizing problems and 3% of the change in internalizing problems at T5.

### Deprivation

**Figure 3.**
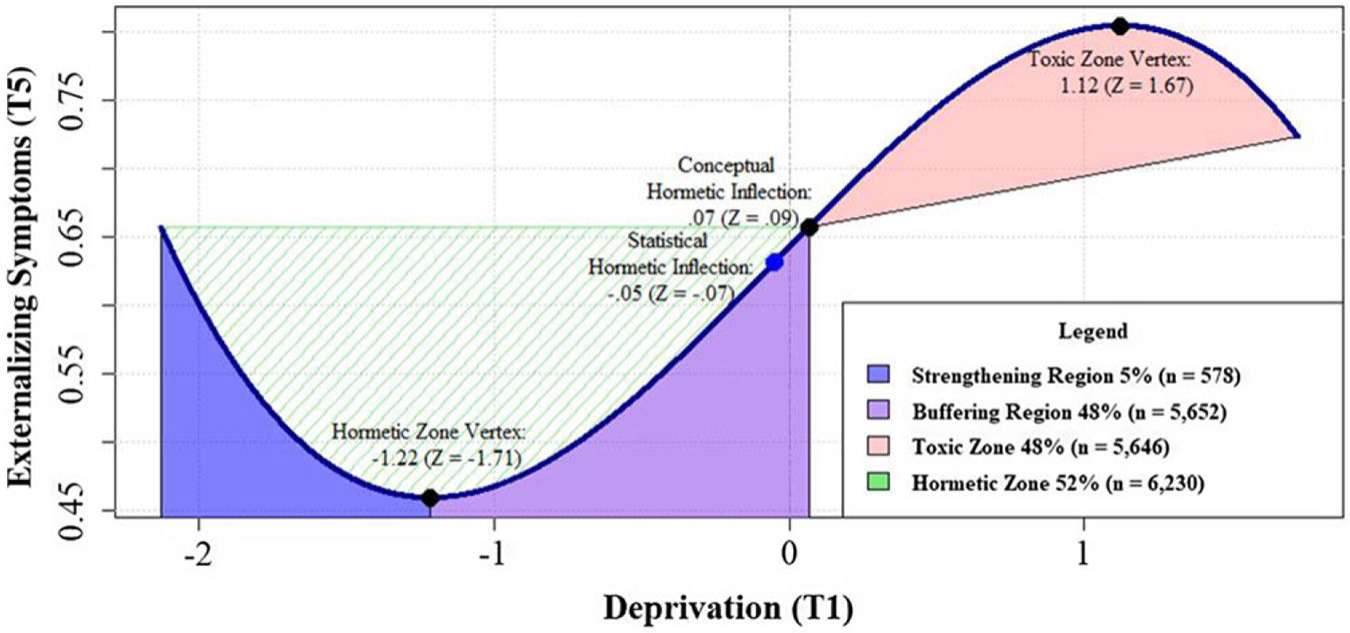
Probing hormesis of deprivation on externalizing symptoms.

The linear model for deprivation predicted change in externalizing problems at T5, *β*_deprivation_ = .08, *CI*[.04, .12], *p* < .001, but not T7 nor either time point of internalizing problems. The addition of the quadratic deprivation path failed to improve model fit (LRT: Δ*χ*^2^(4): 6.66, *p = n.s.*), but the cubic deprivation path did, LRT: Δ*χ*^2^(8): 19.35, *p* < .05, and predicted externalizing problems at T5, *β*_deprivation_^3^ = −.07, *CI*[−.11, −.03], *p* < .01. Follow-up analyses indicated that the conceptual hormetic inflection point (i.e., the transition from hormetic to toxic adversity zones) of the overall cubic curve occurs when deprivation is at .07 units above the mean (*Z* = .09, see Figure 3). The hormetic zone vertex (i.e., the transition from strengthening to buffering adversity regions) for deprivation occurred at −1.22 units below the mean (*Z* = −1.71). Using these thresholds, approximately half of the youths in the total sample were characterized by the hormetic zone, either in the strengthening (5%, *n* = 578) or buffering (48%, *n* = 5,652) regions. In contrast, 48% of the sample (*n* = 5,646) experienced deprivation characteristic of the toxic zone. This model explained 2% of the change in externalizing problems and 3% of the change in internalizing problems at T5.

### Unpredictability

The linear model predicting change in internalizing and externalizing problems from T5-T7 found the unpredictability factor only to be linearly and positively associated with externalizing problems, *β*_unpredict_ = .05, *CI*[.02, .07], *p* < .001. Adding the quadratic and cubic unpredictability terms failed to improve model fit significantly and produced no significant associations. Therefore, the hormetic models were not further probed for the unpredictability dimension.

### Hormetic moderation analysis

**Figure 4.**
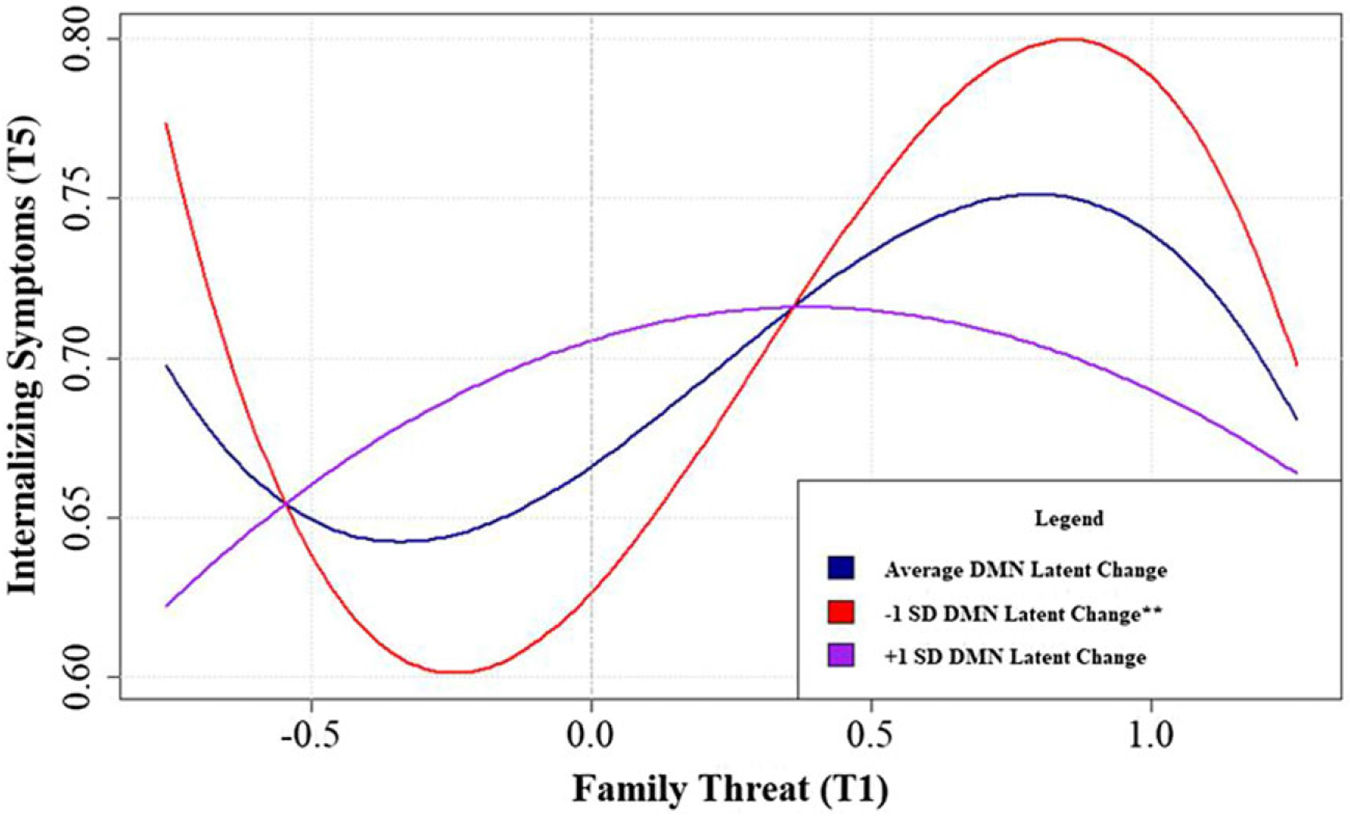
Simple slopes of the cubic family threat X change DMN rsFC interaction predicting youth internalizing problems in the full sample.

Latent change from T1 - T5 within the DMN resting-state functional connectivity (ΔDMN rsFC) was examined as a moderator of the observed hormetic relations between family threat and deprivation on child behavioral outcomes. Only the cubic effect of family threat on internalizing symptoms was significantly moderated by ΔDMN rsFC. This moderating effect persisted after including covariates and correcting for multiple comparisons using the Benjamini-Hochberg approach with a false discovery rate of .05, *β*_THREAT3xΔDMN-LC_ = .10, *CI*[.03, .17], *p*_*corrected*_ = .01. Plotting the simple slopes of the cubic function (See Fig. 4) revealed an overall pattern of effects that lower levels of ΔDMN rsFC intensified the cubic relation between family threat and youth internalizing problems while increases of ΔDMN rsFC flattened this curve.

We further probed the specific moderating effects of ΔDMN rsFC through a series of multi-group analyses. Participants were parsed into conceptually meaningful groups (i.e., strengthening/buffering regions, and toxic/hormetic zones) based on their level of family threat in relation to the inflection points and vertices previously identified on the cubic curve. This approach separates the cubic curve observed in the full sample into smaller sections that can be explored with more conventional linear/quadratic interaction analyses. Specifically, linear interactions were examined in four family threat regions including, strengthening (values less than the hormetic zone vertex [*x* < −32]), buffering (values between the hormetic zone vertex and the conceptual hormetic inflection [*x* = −.31–.33]), rising toxic (values between the conceptual hormetic inflection and toxic vertex [*x* = .34–.75]), and falling toxic (values greater than the toxic vertex [*x* > .75]). Quadratic interactions were examined both in the hormetic (values less than the conceptual hormetic inflection [*x* < .33]) and toxic zones (values greater than the conceptual hormetic inflection [*x* > .33]). Significant within-group interaction paths were compared between groups with a Wald chi-square test. Specific moderating effects and regions of significance were identified using a Johnson-Neyman approach for linear × linear and quadratic × linear effects (Miller et al., 2013), and by plotting simple slopes at +1/−1 *SD* for linear and quadratic associations.

The results of the multi-group interaction analysis found ΔDMN rsFC to significantly moderate *linear* family threat in the strengthening region (*β*_THREATxDMN-LC_ = .31, *CI*[.12, .50], *p* < .01), and *quadratic* family threat in the toxic zone (β_THREAT2xDMN-LC_ = .48, *CI*[.11, .84], *p* < .05). The strengthening region’s linear interaction path was significantly different from the linear interaction paths observed in both the buffering (*W* (1) = 4.38, *p* < .05) and rising toxic regions (*W* (1) = 5.97, *p* < .05). Likewise, the toxic zone quadratic interaction path was significantly different from the quadratic interaction path in the hormetic zone, *W* (1) = 7.83, *p* < .01. These significant between-group tests suggest changes in neural activation patterns have unique effects across the spectrum of family threat that are particularly salient in the strengthening region and the toxic zone.

**Figure 5.**
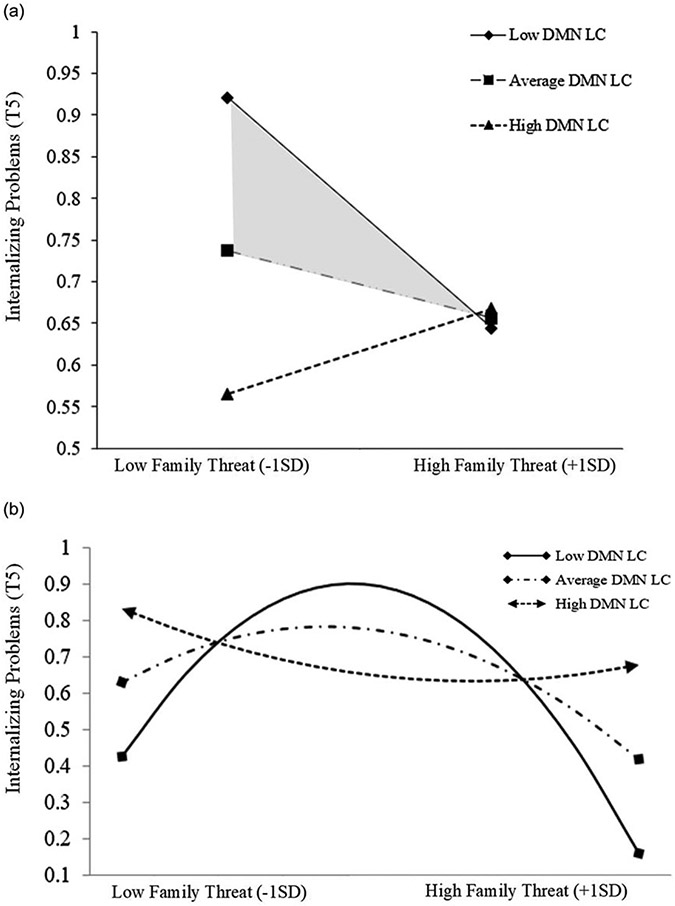
(***a***) Probing interaction by DMN LC in the strengthening zone. (*b*) Simple slopes of the family threat^2^ x DMN LC predicting internalizing problems in the toxic zone.

Probing the within-group linear and quadratic effects reflected the same exacerbating role of low ΔDMN rsFC observed during the initial cubic moderation analysis (Fig. 4). Within the strengthening region, the negative linear relation between family threat and internalizing symptoms became more negative as ΔDMN rsFC lessened (Fig. 5a). In the toxic zone, the concavity in the quadratic relation between family threat and internalizing symptoms increased as ΔDMN rsFC decreased (Fig. 5b). In other words, lower ΔDMN rsFC changed its effect in different family risk contexts. Specifically, on the one hand, lower ΔDMN rsFC was associated with increases in the strength of the hormetic effect of family threat on the development of internalizing problems in the strengthening region (family threat became *more* beneficial at low ΔDMN rsFC; See Fig. 5a). On the other hand, lower ΔDMN rsFC was linked with the potentiation of the harmful effects of family threat in the toxic zone (low ΔDMN rsFC increased the harm of family threat; See Fig. 5b).

**Figure 6.**
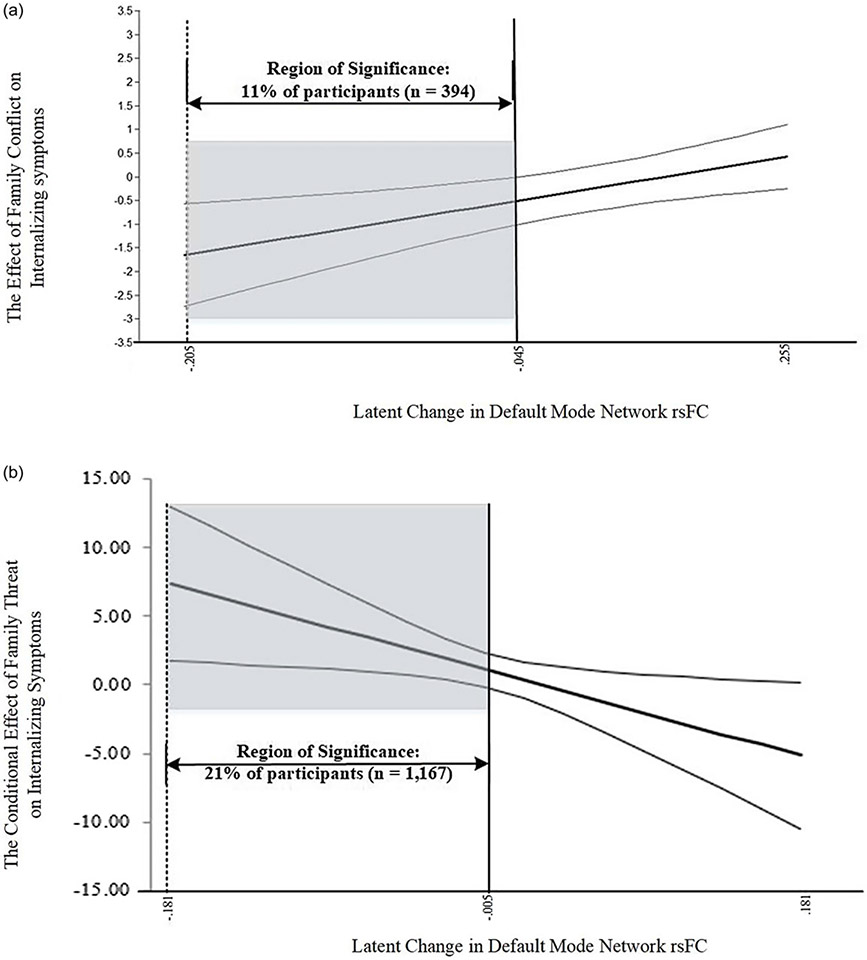
(***a***) Johnson-Neyman plot of cubic interaction by DMN LC strengthening zone. (***b***) Johnson-Neyman plot of family threat across values of the DMN LC when X is fixed at its mean (0).

Johnson-Neyman analyses indicated that the moderating effect of ΔDMN rsFC influenced 1,561 (14%) of youths in the overall sample (strengthening *n* = 394, toxic *n* = 1,167). Moreover, areas at which the influence of the moderator became significant (area of significance; AoS) were identified as ΔDMN rsFC values below −.046 within the strengthening region (linear interaction; See Fig. 6a) and −.005 in the toxic zone (quadratic interaction; See Fig. 6b). The negative ΔDMN rsFC values characterizing each AoS indicate *decreases* in DMN rsFC coherence from T1 to T5 (i.e., lower correlation among DMN regions). In fact, post-hoc analyses found that baseline and T5 DMN rsFC means, variances, and the latent change scores were statistically equivalent across groups (strengthening, buffering, and rising/falling toxic regions; Δ*χ*^2^(12) = 5.95, *p* = .92). However, youths in an AoS (either strengthening region or toxic zone) experienced *higher* baseline levels of DMN rsFC (ΔM_DMNrsFC-T1_ = .06; *W* (1) = 240.01, *p* < .001) and *lower* DMN rsFC at T5 (ΔM_DMNrsFC-T5_ = −.04; *W* (1) = 158.94, *p* < .001) compared to the remainder of the sample. Additionally, the AoS youths were characterized by a negative ΔDMN rsFC mean (ΔDMN rsFC_AOS_ = −.08) compared to the positive ΔDMN rsFC mean found in the overall sample (ΔDMN rsFC_NONSIG_ = .02; *W* (1) = 1,608.34, *p* < .001). In summary, the accentuating effect of ΔDMN rsFC on the association between family threat and youth internalizing problems is significant for youths experiencing *reductions* in DMN rsFC over the two-year period (from Time 1 to Time 5).

## Discussion

The developmental psychopathology perspective offers a rich theoretical framework for studying the origin, course, and mechanisms of resilience. A central focus of developmental psychopathology research is to understand how typical versus atypical development emerges under both normal and abnormal conditions (Cicchetti & Curtis, 2015). A critical yet unanswered question is how human resilience unfolds as a developmental process in response to, rather than despite, adverse experiences (Oshri et al., 2022). The hormesis model, widely used in toxicology, explains the salubrious and strengthening effects of environmental conditions on organisms’ development. However, what constitutes the ‘environment’ in toxicological or biomedical fields differs from its application to human development (Oshri, 2023). This paper aims to test the utility of applying the hormesis model to DP perspective for studying the development of the human resilience process. We aimed to explore what environmental conditions and dimensions of psychosocial adversity may result in strengthening effects and/or risk for developing psychopathology among youth, and how brain-by-development context fosters interindividual variability in hormetic responses.

The current study used a longitudinal sample of 11,878 youth to examine the effect of dimensions of psychosocial adversity, including threat, deprivation, and unpredictability, on change in internalizing and externalizing symptoms of psychopathology. Further, we examined the resilience process to these environmental adversity dimensions by testing the psychosocial hormetic model of resilience. Specifically, we aimed to test whether these dimensions of adversity are related to strengthening, buffering, and toxic effects from stress. This allowed us to examine the hypotheses that resilience may also emerge in response to low-to-moderate stress levels and that this effect may be moderated by resting-state neural context. Results showed that threat was positively related to both increases in internalizing and externalizing symptoms, whereas deprivation and unpredictability were positively associated only with externalizing problems. A nonlinear examination of the hormesis model showed that threat was cubically related to change in internalizing symptoms. Deprivation, in turn, was cubically related to externalizing symptoms, and unpredictability only showed linear associations with internalizing and externalizing symptoms. When testing the within-DMN rsFC change as a moderator of the hormetic effect of family threat on youth internalizing problems, we found that a decline in DMN rsFC (from T1 to T5) altered this association in a *“for better-or-worse”* fashion. That is, reductions in DMN rsFC amplified both the beneficial effects of low-to-moderate levels of adversity and also the deleterious effects of high levels of family threat on internalizing problems.

The results that threat was positively related to increased risk for psychopathology corroborate the emotional security theory (Cummings et al., 2015). Furthermore, this theory has been further advanced to include multiple types of conflict in the family beyond interparental conflict (Cummings et al., 2015). Because the family environment is critical in shaping youth emotional regulation during childhood and adolescence, the effect of being brought up in a conflictual family climate is salient for adolescent psychopathology outcomes. The emotional security theory argues that in the face of threat, regulatory response patterns in children are forming and inform later coping responses to future threats from the environment (Cummings & Davies, 2010). Accordingly, the more children and youth are exposed to increased levels of family conflict over time, the more they are likely to solidify dysregulated emotional responses to stressful environments, which increases their vulnerability to affective symptoms such as internalizing problems.

Likewise, our results revealed a cubic effect in which low-to-moderate levels of family conflict led to a reduction in the change of internalizing psychopathology in adolescence. These findings support research in which interparental conflict was found to be congruent with the psychosocial model for hormesis. For example, in a prospective study, Davies and colleagues (Davies et al., 2022) showed a nonlinear (quadratic) association between children’s emotional reactivity and behavioral dysregulation. In this study, minimal levels of family conflict were linked to less emotional risk, whereas high levels of family conflict were related to children’s risk for psychopathology (Davies et al., 2022). Our study adds to this literature by characterizing the cubic association between threat and internalizing problems in adolescence and showing to what extent this effect strengthens and buffers against risk.

The present study tested hormetic moderation by change in DMN rsFC to investigate whether variability in hormetic effects partly stems from individual differences in DMN rsFC changes over time. Results showed that a change in DMN rsFC moderated the link between family threat and later internalizing problems. Specifically, we found that a significant reduction in DMN rsFC from Time 1 to Time 5 increased the strength of the hormetic association between family threat and internalizing problems (i.e., low-to-moderate family conflict became more protective). This suggests that among those who started high (Time 1) in DMN rsFC, a decline in this metric was conducive to hormetic neuroplasticity in this age range. In contrast, individuals who started relatively low in DMN rsFC at Time 1 tended to show increases in DMN rsFC at Time 5 and did not show a hormetic effect.

Moreover, individuals who evinced a reduction in DMN rsFC have shown differential risk for psychopathology across varying levels of family threat in a *for better-or-worse* pattern. While at low-to-moderate levels of threat, these families showed strengthening effects, at high levels of family threat, these individuals experienced the greatest levels of psychopathology and fell into the toxic hormetic zone (See Fig. 5a,b). Such a change from strengthening to vulnerability outcomes at a specific pattern of change of DMN rsFC across varying levels of family threat may be consistent with biological sensitivity to context theory (Ellis & Boyce, 2008; Ellis et al., 2005), supporting earlier work testing biological sensitivity to context using neurobiological data (Gard et al., 2018; Liu et al., 2021; Oshri, 2023). Thus, this study may be the first to indicate a biological sensitivity effect to hormesis.

The finding that hyperconnectivity reflects higher sensitivity to the environment is consistent with earlier work on the role of the DMN in brain-behavior associations. As noted above, heightened DMN rsFC reflects a high within-network DMN engagement history. Given that coordinated switching between DMN and other task-active networks (e.g., SAL or FPN) supports internal and external modes of processing and attention, heightened DMN rsFC has been linked to some youths’ difficulty over time in redirecting neural resources from internally directed thoughts to external stimuli for adaptive task performance. Hyperconnectivity may, in turn, have placed them at increased risk for psychopathology (Whitfield-Gabrieli & Ford, 2012). This interpretation may partially explain our findings, particularly for individuals in the high family threat context. We extend this model by considering stress as a key contextual moderator. We posit that individuals with higher vs. lower DMN rsFC at Time 1 may reflect relatively more advanced maturation in this network. For individuals who start high (T1) and decrease (T5) and experience low family threat, this advanced maturation may support adaptive neuroplasticity and be more conducive to developing coping strategies, likely via inter-network coordination. However, when family threat is high and, presumptively, stress levels are also high, overall network functioning may be overwhelmed, especially in frontal regions facing competing demands (e.g., attention towards external, salient [threatening] contextual cues in the environment vs. problem-solving, developing coping strategies). Depleting these PFC resources may thus expose vulnerability to task-switching difficulties involving the DMN and, ultimately, a greater risk for the development of psychopathology.

The effect of deprivation on externalizing problems was found in the present study to be positive (linearly), supporting a large body of research on the effects of deprivation in children’s rearing environment on child risk for the development of externalizing problems (Bolland et al., 2016; Lopez-Vergara et al., 2016). In contrast to our expectations, no significant associations existed between children’s exposure to deprivation and changes in internalizing problems. The finding corroborates studies showing that youth who experience deprivation in the form of SES hardship and lack of parental supervision are more likely to develop externalizing symptoms such as conduct and delinquency problem behaviors (Borawski et al., 2003; Ryan et al., 2013). These fit in particular to preadolescence, a developmental transition in which neglect at the family level changes from parental supervision to parental monitoring, which is highly associated with conduct problems (Ryan et al., 2013). In contrast to threat, deprivation did show a cubic association with externalizing symptoms, suggesting that 52% evinced hormesis. The results revealed that 5% of the youth fell into the strengthening zone, and 48% to the buffering zone, suggesting that very low levels of family deprivation yielded some strengthening effects that reduced the risk for later psychopathology.

Studies on the impact of unpredictability on youth development have established a robust link to the development of psychopathology (Glynn & Baram, 2019; Hunt et al., 2023). Our models showed that unpredictability has no hormetic effects but a linear impact on the risk of internalizing and externalizing problems. These findings corroborate life history theory and research on the developmental implications of unpredictability. Accordingly, life history theory suggests that unpredictability is particularly stressful for youth and may trigger faster life history strategies or energy allocation priority to behaviors that accelerate physical and cognitive growth and reproductive success, including aggressive and delinquent behavior (Ellis et al., 2009). Additionally, plasticity tends to be most pronounced in childhood and adolescence (Fu & Zuo, 2011). However, in an unpredictable environment, there is less opportunity for experience-dependent learning, which is necessary for hormetic effects (Oshri et al., 2022) and resilience-building capacity. Such inconsistent environmental input undermines learning and the required neuroplasticity (Kolb & Gibb, 2014) to respond, adapt, and prepare for future adverse environments.

As demonstrated in this paper, in a specific dimension of an adverse environment, youth may exhibit variability in developmental outcomes that cannot be thoroughly tested without nonlinear models. This is due to the potential modification of the path from the independent variables representing the experience of adversity that significantly influences human development. Sensitive and critical periods are fundamental mechanisms driving individual variability in response to environmental inputs, including adversity (Hartley & Frankenhuis, 2020). This study partially examined the concept of timing in hormetic plasticity. We found that experiences of threat and deprivation had more pronounced hormetic effects in relation to psychopathological outcomes at earlier ages than later. This aligns with the idea that the timing of developmental outcomes can be altered by neural circuitry organization and function (Gabard-Durnam & McLaughlin, 2020). Research in settings like orphanages has documented the harmful impact of depriving environments in adoptive institutions during infancy on emotional and psychological outcomes (Gunnar & Bowen, 2021). Interestingly, there is limited research on the sensitive periods for resilience-building plasticity. It remains unclear at what developmental stages low-to-moderate adversity might foster growth and resilience.

**Figure 7.**
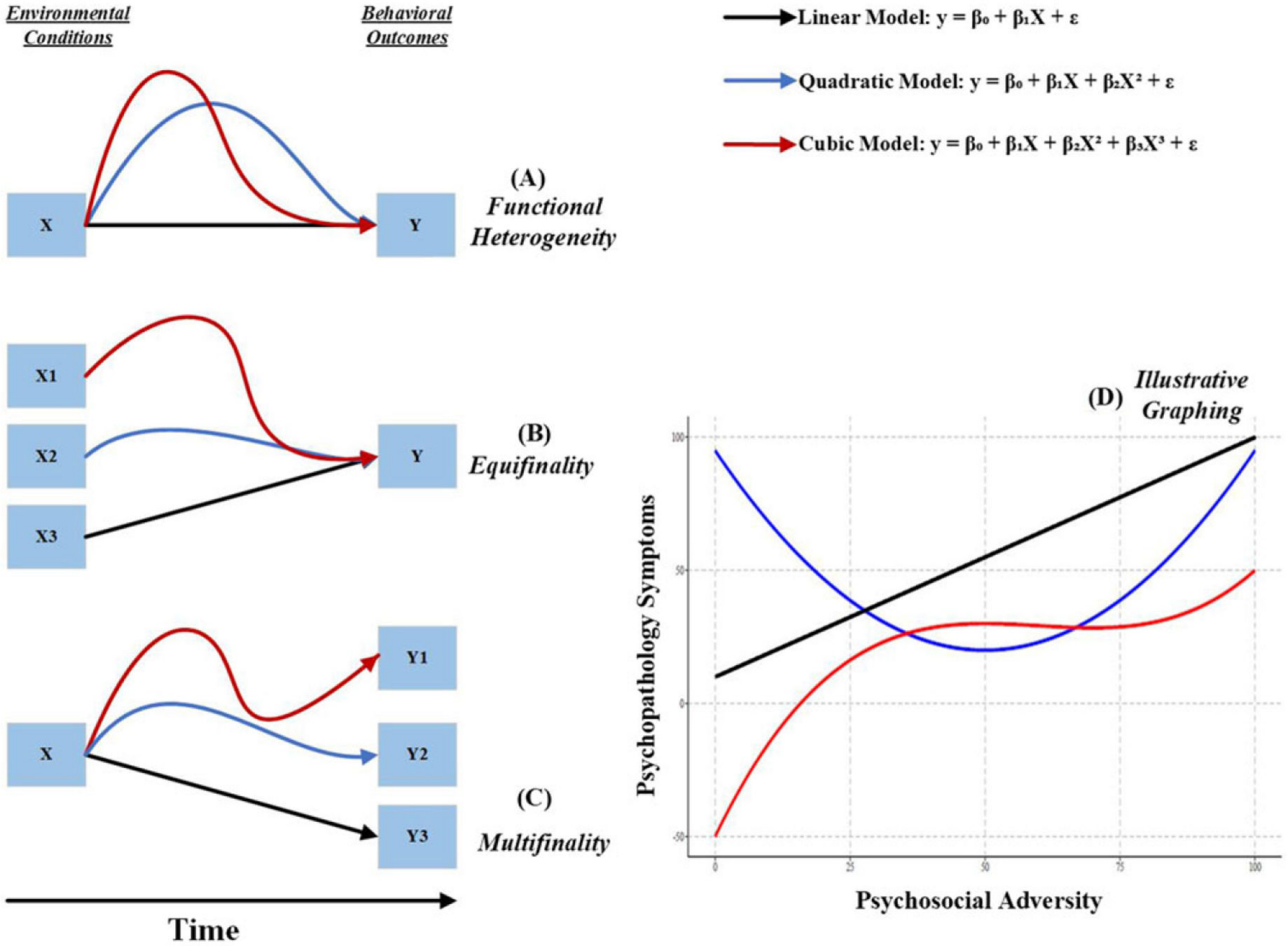
Theoretical visualization of multifinality and equifinality.

Further study is needed to understand how the concepts of multifinality and equifinality relate to hormesis, resilience, and risk in the development of psychopathology. In Figure 7, we provide a theoretical presentation of the connection between a given adverse environmental condition (denoted as X1) and a behavioral outcome symptomatic of psychopathology (Y1). In the first illustration, we show that a given developmental connection between the environment and a behavioral outcome can manifest in multiple ways. It can be linear, and nonlinear, suggesting that the statistical modeling of the development of psychopathology can be missed, regardless of other moderating and mediating variables in the prediction models. This brings the discussion to multifinality and equifinality in developmental science and resilience.

First, consistent multifinality, we can hypothesize an adverse environmental condition (*X*_1_) to the development of multiple behavioral outcomes (Y_1_–Y_3_) by nonlinear effects (Cicchetti & Toth, 2009). The principle of multifinality suggests that while some children may exhibit resilience or even positive adaptation after exposure to potentially threatening environments, others may not, highlighting the variability in outcomes following similar experiences. This variability in the effects of adverse environments is surely affected by moderating and mediating mechanisms, however it can also stem from the fact that the a*dverse condition itself promoted differential risk* to behavioral outcomes. Such differences underscore the potential hormetic effects of early experiences, where low-level exposure to stressors can enhance resilience, and reduce risk for psychopathology in some, but not all, individuals. This phenomenon contributes to the observed interindividual variability in the risk of developing psychopathology, challenging linear analyses to fully capture these dynamics.

Similarly, the concept of equifinality, where different dimensions of environmental adversity (X1, X2, X3) lead to a similar developmental outcome (Y), underscores the need for considering nonlinear pathways. This approach recognizes that despite varied adverse experiences, similar outcomes may arise, a process potentially moderated by exposure to multilevel influences of risk and protective factors among youth. Identifying the mechanisms that promote the process of resilience is crucial for developing effective prevention and intervention strategies. To clarify, hormesis provides a framework for understanding the nuanced ways that stressors can influence development. It posits that mild stressors can foster adaptive and strengthening responses, thereby contributing to resilience. This concept helps explain the observed interindividual variability in developmental outcomes, aligning with multifinality and equifinality by illustrating how different levels of stressor exposure and individual responses can lead to varied outcomes from similar or different environmental conditions.

### Strengths and limitations

This study leverages multilevel prospective data from 11,878 youths aged 10 to 13, including neuroimaging and survey data. We based our models on a brief survey measure to derive the family threat variable, employing an advanced SEM measurement approach for this purpose. Further, we derived the constructs of deprivation and unpredictability from multiple levels and reporters. In this framework, deprivation is a latent factor, while unpredictability is analyzed through second-order factor analysis. Thus, the first limitation to consider is the temporal sequence from independent to dependent variables. Notably, the unpredictability variable, obtained from data at T5, does not align temporally with other stressors. Temporal sequence also impacted the composition of the threat factor that was limited to a selection of variables in the family conflict scale rather than a more holistic collection of variables. However, all psychopathology outcomes were measured prospectively after the independent variables. An additional limitation is that the ABCD sample represents a community cohort that was not explicitly sampled to include families from high-adversity environments. Nonetheless, our data indicate considerable exposure to SES hardship (29.7% with income < $40K), revealing significant variability in risk and adversity. Lastly, because we weren’t aware of previous studies on the hormetic effects in the development of psychopathology and in a brain-by-development context, some analyses were exploratory and guided by data-driven approach. For example, we tested multiple brain regions associated with DMN to test the hormesis hypothesis. However, these data are public, and our syntaxes are available for transparency and future replication efforts (https://github.com/YouthDevelopmentInstituteUGA?tab=repositories).

A central neurobiological marker in this study is DMN rsFC, defined here as the mean correlation value across all within-DMN connections. The DMN comprises (Gordon et al., 2020; Raichle et al., 2001), multiple regions, including the bilateral medial parietal cortex, medial and superior PFC, angular gyrus, medial and lateral temporal lobes, and cerebellum. As such, DMN rsFC reflects the average functional association among all these regions. Studies examining DMN rsFC at the network level have demonstrated numerous associations with normative and non-normative cognitive functioning (Whitfield-Gabrieli & Ford, 2012). While the DMN is indeed identifiable during resting-state fMRI as a single, unitary network, recent work indicates the presence of more nuanced organizational and functional architectures—subnetworks—within the DMN as well as other resting-state functional networks (Doucet et al., 2011; Uddin et al., 2009). This is a limitation because it narrows our ability to fully utilize the rich heterogeneity in brain function found within and across individuals in the ABCD Study broadly and, more specifically, assess what role subnetworks may play in the complex associations between early-life stress, risk for psychopathology, and resilience. As such, future work in this area should conceptualize and examine brain networks functioning at multiple levels of operation, from regional brain activity to subnetworks to networks, and develop user-friendly methods allowing researchers to do so in large, publicly available datasets.

## Figures and Tables

**Table 1.** Items factor loadings for family unpredictability, deprivation, and threat

Factors & indicators	Factor loadings	
Family unpredictability	First- Order	Higher- Order
**Primary caregiver – unpredictability**		.89
*My moods or feeling change suddenly*	.38	
*People think i am disorganized*	.61	
*I have trouble managing my money or credit card*	.54	
**Secondary caregiver – unpredictability**		.56
*My moods or feeling change suddenly (about partner)*	.46	
*People think i am disorganized (about partner)*	.66	
*I have trouble managing my money or credit card (about partner)*	.59	
**Family disorder**		.58
*We are generally very neat and orderly*	.83	
*Family members make sure their rooms are neat*.	.82	
*Each person’s duties are clearly defined in our family*	.48	
*Dishes are usually done immediately after eating*	.62	
**Deprivation**		
*Family material deprivation scale*	.42	
*Parental education*	.76	
*Income-to-poverty ratio*	.83	
*Neighborhood areas deprivation index*	.57	
*Marital status*	.54	
*Parental monitoring – average number of family dinners per week*	.18	
**Family threat**		
*We fight a lot in our family*	.81	
*Family members rarely become openly angry (r)*	.46	
*Family members sometimes get so angry they throw things*	.74	
*Family members hardly ever lose their tempers (r)*	.59	
*Family members often criticize each other*	.63	
*Family members sometimes hit each other*	.74	
*Family members often try to one-up or outdo each other*	.50	

*Note.* All factor loadings *p* < .01.

**Table 2.** Longitudinal cubic regression parameters on internalizing and externalizing symptoms

Model	2-Year Assessment						3-Year Assessment					
	Internalizing Problems			Externalizing Problems			Internalizing Problems			Externalizing Problems		
	1	2	3	1	2	3	1	2	3	1	2	3
Threat												
Intercept	**−.95 (−1.78)** ***	**−.95 (−1.77)** ***	**−.97 (−1.81)** ***	.11 (.12)	.11 (.11)	.09 (.10)	−.12 (−0.16)	−.13 (−.16)	−.13 (−.17)	**.37 (.17)** *	**.38 (.18)** *	**.38 (.18)** *
Youth Sex	**.16 (.58)** ***	**.16 (.58)** ***	**.16 (.58)** ***	**.05 (.11)** ***	**.05 (.11)** ***	**.05 (.11)** ***	**.10 (.25)** ***	**.10 (.25)** ***	**.10 (.25)** ***	**.05 (.04)** ***	**.05 (.04)** ***	**.05 (.04)** ***
Youth Age	**.05 (.01)** ***	**.05 (.01)** ***	**.05 (.01)** ***	**.04 (.01)** ***	**.04 (.01)** ***	**.04 (.01)** ***	.01 (.001)	.01 (.001)	.01 (.001)	.01 (.00)	.01 (0.00)	.01 (.00)
Family Income	.01 (.01)	.01 (.011)	.01 (.01)	**−.08 (−.04)** ***	**−.08 (−.04)** ***	**−.08 (−.04)** ***	**.04 (.02)** ***	**.04 (.02)** ***	**.04 (.02)** ***	**−.03 (.01)** *	**−.03 (−.01)** *	**−.03 (−.01)** *
T1 Family Threat	.01 (.02)	.01 (.03)	**.05 (.15)** *	**.08 (.14)** ***	**.08 (.13)** ***	**.11 (.18)** ***	−.01 (−.01)	−.01 (−.01)	.001 (.003)	**.03 (.02)** *	**.04 (.03)** ***	**.05 (.04)** *
T1 Family Threat^2^		−.004 (−.02)	.033 (.13)		.01 (.02)	.03 (.08)^ϯ^		.002 (.01)	.01 (.03)		**−.03 (−.03)** *	−.02 (−.02)
T1 Family Threat^3^			**−.07 (−.17)** *			−.05 (−.07)			−.01 (−.02)			−.02 (−.01)
LRT: Δ*χ*^2^ (df)		**9.53 (4)** *	**9.68 (4)** *		**9.53 (4)** *	**9.68 (4)** *		**9.53 (4)** *	**9.68 (4)** *		**9.53(4)** *	**9.68 (4)** *
Deprivation												
Intercept	**−.95 (−1.77)** ***	**−.90 (−1.67)** ***	**−.90 (−1.67)** ***	−.03 (−.04)	−.07 (−.08)	−.08 (−.08)	−.07 (−.09)	−.04 (−.06)	−.04 (−.06)	.34 (.16)^ϯ^	.32 (.15)^ϯ^	.32 (.15)^ϯ^
Youth Sex	**.15 (.57)** ***	**.15 (.57)** ***	**.15 (.57)** ***	**.05 (.10)** ***	**.05 (.10)** ***	**.05 (.10)** ***	**.10 (.25)** ***	**.10 (.25)** ***	**.10 (.25)** ***	**.05 (.25)** ***	**.05 (.04)** ***	**.05 (0.043)** ***
Youth Age	**.05 (.01)** ***	**.05 (.01)** ***	**.05 (.01)** ***	**.04 (.01)** ***	**.04 (.01)** ***	**.04 (.01)** ***	.01 (.001)	.01 (.001)	.01 (.001)	.01 (.001)	.01 (.00)	.01 (.00)
Family Income	.01 (.01)	−.001 (−.001)	.00 (.00)	−.03 (−.01)	−.01 (−.01)	−.01 (−.004)	.02 (.01)	.01 (.004)	.01 (.01)	−.01 (−.003)	−.01 (−.002)	−.01 (−.001)
T1 SES Deprivation	.001 (.004)	−.02 (−.05)	−.003 (−.01)	**.08 (.13)** ***	**.10 (.15)** ***	**.14 (.22)** ***	−.03 (−.05)	−.04 (−.07)	−.01 (−.03)	.02 (.01)	.03 (.02)	.05 (.03)^ϯ^
T1 SES Deprivation^2^		−.02 (−.06)	−.03 (−.09)^ϯ^		.02 (.03)	−.01 (−.02)		−.01 (−.02)	−.02 (−.05)		.01 (.01)	−.004 (−.003)
T1 SES Deprivation^3^			−.02 (−.04)			**−.07 (−.07)** ***			−.03 (−.04)			−.03 (−.01)
LRT: Δ*χ*^2^ (df)		6.66 (4)	**19.35 (8)** *		6.66 (4)	**19.35 (8)** *		6.66 (4)	**19.35 (8)** *		6.66 (4)	**19.35 (8)** *
Unpredictability												
Intercept							−.16 (−.22)	−.16 (−.22)	−.17 (−.23)	.33 (.16)^ϯ^	.34 (.16)^ϯ^	.32 (.15)^ϯ^
Youth Sex							**.10 (.27)** ***	**.10 (.27)** ***	**.10 (.27)** ***	**.05 (.05)** ***	**.05 (.05)** ***	**.05 (.05)** ***
Youth Age							.01 (.001)	.01 (.001)	.01 (.001)	.01 (.001)	.01 (.001)	.01 (.001)
Family Income							**.04 (.02)** **	**.04 (.02)** ***	**.04 (.02)** **	**−.03 (−.01)** *	**−.03 (−.01)** *	**−.03 (−.01)** *
T5 Unpredictability							−.02 (−.20)	−.02 (−.19)	−.01 (−.17)	**.05 (.20)** ***	**.05 (.22)** ***	**.06 (.24)** ***
T5 Unpredictability^2^								−.001 (−.06)	.02 (.89)		−.01 (−.19)	.03 (.48)
T5 Unpredictability^3^									−.02 (−2.71)			−.04 (−1.93)
LRT: Δ*χ*^2^ (df)								.39 (2)	2.94 (4)		.39 (2)	2.94 (4)

*Note.* Table format β (b). LRT = MLR Scaling Corrected Likelihood Ratio Test comparing current model to the significant former. *** *p < .001,* ** *p < .01,* * *p < .05, ^ϯ^ p < .1.*
